# Gender difference in adiponectin associated with cardiovascular mortality

**DOI:** 10.1186/s12881-015-0187-9

**Published:** 2015-06-12

**Authors:** Urban Alehagen, Emina Vorkapic, Liza Ljungberg, Toste Länne, Dick Wågsäter

**Affiliations:** Division of Cardiovascular Medicine, Department of Medicine and Health Sciences, Faculty of Health Sciences, Linköping University, Department of Cardiology UHL, County Council of Östergötland, Linköping, Sweden; Division of Drug Research, Department of Medical and Health Sciences, Faculty of Health Sciences, Linköping University, Linköping, Sweden

**Keywords:** Genotypes, Gender, Prognosis

## Abstract

**Background:**

It is important to identify cardiovascular diseases in patients at high risk. To include genetics into routine cardiological patients has therefore been discussed recently.

We wanted to evaluate the association between high-molecular weight adiponectin and cardiovascular risk, and secondly in the same population evaluate if specific genotype differences regarding risk could be observed, and thirdly if gender differences could be seen.

**Method:**

Four hundred seventy-six elderly participants recruited from a rural community were included. All participants underwent a clinical examination, echocardiography, and blood sampling and the single nucleotide polymorphism (SNP) (rs266729) of adiponectin was analysed. Follow-up time was 6.7 years.

**Results:**

Those with high serum concentration of adiponectin had a more 2 fold increased cardiovascular risk, and it might be that females exhibits even higher risk where a more than 5 fold increased risk could be seen. The result could be demonstrated even in a multivariate model adjusting for well-known clinical risk factors. However, as the sample size was small the gender differences should be interpreted with caution. In the genotype evaluation the C/C carriers of the female group had a more than 9-fold increased risk of cardiovascular mortality, however the confidence interval was wide. Such genotype difference could not be found in the male group.

**Conclusion:**

High level of adiponectin was associated with increased cardiovascular risk. Also a gender difference in the genotype evaluation could be seen where the C/C carriers obtained higher risk in the female group but not in the male group. Thus, in order to identify patients at risk early, genetic analyses may add to the armamentarium used in the clinical routine. However, information should be regarded as hypothesis generating as the sample size was small and should stimulate further research in individualized cardiovascular prevention and treatment.

## Background

Adiponectin is a peptide hormone and secreted from adipose tissue. It has a multitude of actions in the body, and has been shown to have insulin-sensitizing effects [1], anti-atherosclerotic effects [2], but also anti-inflammatory effects [3]. In obese patients the circulating levels of adiponectin are reduced [4]. Interesting, but contradictory data of the role of adiponectin in the cardiovascular area have been published. Li et al., report that in patients with coronary artery disease, a low plasma concentration of adiponectin is associated with higher cardiovascular risk [5]. From a nested case-control study of more than 18,200 males Pischon et al. reported that a high plasma concentration of adiponectin was associated with a lower risk of myocardial infarction in persons without coronary artery disease [6]. However, Hascoet et al. report a higher cardiovascular risk in those with a high serum concentration of adiponectin from a population with established coronary artery disease, but also in controls [7]. Also, the Health ABC Study, Poehls et al. reported that in elderly healthy persons a higher concentration of adiponectin was associated with higher cardiovascular risk [8], and in elderly men without diagnosed cardiovascular disease Wannamethee et al. reported a higher cardiovascular risk in those with high plasma concentration of adiponectin [9]. In an effort to evaluate a larger material Wu et al. reported from a meta-analysis including 14 different studies a strong association between high concentration of adiponectin and all-cause mortality, but also from eleven different studies in a meta-analysis a strong association between high concentration of adiponectin and cardiovascular mortality [10]. From a study of almost 700 female patients Mackey et al. could report an association between high concentration of adiponectin and coronary heart disease in those with diabetes, but not among those without diabetes [11]. In elderly persons with atrial fibrillation, a higher concentration of adiponectin was reported by Macheret et al. in a study of more than 3000 individuals [12]. In an interesting study performed in dogs, Damoiseaux C et al. demonstrated a higher adiponectin concentration in dogs with dilated cardiomyopathy, compared to healthy dogs of the corresponding age [13]. From Portugal, Fontes-Carvalho et al. reported that females, but not males with high concentration of adiponectin had increased risk for diastolic impairment of the heart [14]. From three surveys of healthy persons Karakas et al. could not report any associations between serum level of adiponectin and risk coronary heart disease [15]. Thus a complex picture is reported regarding a possible association between adiponectin and cardiovascular risk. However, in the vast majority of reports total adiponectin is analyzed, where only a minority of articles report on high-molecular-weight adiponectin, the biologically active part [16].

From the literature it is also evident that different genotypes of adiponectin could associated with cardiovascular risk. However, various researchers have investigated the role of the adiponectin gene in the risk of coronary artery disease with inconsistent results [17–19], and different single nucleotide polymorphisms (SNPs) of adiponectin have different associations with coronary artery disease risk [20–22]. The SNP rs266729 of adiponectin has been reported with increased risk of coronary artery disease in some reports [17, 23, 24], whereas Cheung et al. could not report any association between rs266729 and risk of coronary artery disease either in males nor in females [25]. Pileggi et al. reported that that the G allele of rs266729 was associated with increased risk of heart failure as evaluated from a population of more than 2300 Italian participants of which half had heart failure [26]. Thus, there is evidence that rs266729 could give prognostic cardiovascular information based on the literature.

The primary aim of this study was therefore to evaluate a possible association of cardiovascular death with expression of high molecular weight of adiponectin and rs266729 gene polymorphism. The secondary aim was to evaluate if the associations are gender specific.

## Methods

### Patient population

The study population consisted of 476 apparently healthy individuals (men: 242; females: 234) with a mean age of 77.0 years (range:18 years) living in a rural municipality in the south-east of Sweden and invited to participate in this project. They were all part of a longitudinal epidemiological study focusing on cardiovascular risk factors [27]. All the participants in that study were invited to participate in the present sub-study conducted from 13^th^ January 2003 through 18^th^ June 2005. All participants underwent a clinical examination, ECG recording, and Doppler-echocardiography, and blood samples were collected. New York Heart Association (NYHA) functional class classification (The NYHA functional class grades how a patient with heart disease experiences symptoms of tiredness, breathlessness or chest pain and is graded from I-IV, where IV is symptoms experienced while at rest) was evaluated by the including physician based on the patient information. All participants gave their informed consent, and the study was conducted in accordance with the Declaration of Helsinki principles. The study protocol was approved by the Regional Ethical Review Board of Linköping, Sweden (Dnr 95044). The mortality information was obtained from the National Board of Health and Welfare in Sweden, which registers all deaths, or from autopsy reports.

### Morbidity

Diabetes mellitus was defined as a previous diagnosis with on-going treatment, or a blood glucose ≥ 7 mmol/L. Hypertension was defined as a previous diagnosis with on-going treatment or a blood pressure ≥ 140/90 mmHg at rest. Ischemic heart disease was defined as a history of angina pectoris/myocardial infarction or ECG-verified myocardial infarction. Heart failure was defined as a previous diagnosis with on-going treatment, or symptoms/signs of heart failure and objective demonstration of reduced cardiac function in terms of impaired cardiac function on echocardiography, or increased plasma concentration of proBNP 1–76.

### Echocardiography

Echocardiography examinations were performed on (Accuson XP-128c) with the patients in a supine left position. Values for systolic function expressed as left ventricular ejection fraction (EF), were categorized into four classes with interclass limits 30 %, 40 % and 50 %. Normal systolic function was defined as EF ≥ 50 %.

### Biochemical analyses

All blood samples were collected in pre-chilled plastic Vacutainer tubes (Terumo EDTA K-3, Terumo Europe N.V., Leuven, Belgium) obtained while the patients were at rest in a supine position. Plasma was prepared by centrifugation at 3000 g for 10 min at 4 °C. All samples were stored at—70 °C until analysis, and none of the samples were thawed more than twice.

ProBNP 1–76 (NT-proBNP) was measured using the Elecsys 2010 platform (Roche Diagnostics, Mannheim, Germany). The total coefficient of variation was 4.8 % at 26 pmol/L and 2.1 % at 503 pmol/L (*n* = 70) at our laboratory.

### HMW-Adiponectin

High molecular weight form of adiponectin concentrations were measured in serum using Quantikine ELISA kit according to the manufacturer’s instructions (R&D Systems, Abingdon, United Kingdom). In brief, serum samples were diluted 1:100 and added to the mouse monoclonal capture antibody precoated wells for three hours. After washing, high molecular weight conjugate was added for one hour, which was followed by washing, and thereafter substrate solution was added for 30 min. After addition of stop solution, optical density of each well was determined using a microplate reader set to 450 nm with wavelength correlation set to 540 nm.

### Genotype determination

Genomic DNA was isolated from peripheral blood using QIAmp DNA Mini Kit (QIAGEN, Hilden, Germany), following the manufacturer’s instructions. The 7500 Fast Real-Time PCR system (Applied Biosystems, CA, USA) was used for analysis of adiponectin (rs266729) using a TaqMan SNP genotyping assay mixed with TaqMan Universal PCR Master mix II (Applied Biosystems).

### Statistical methods

Descriptive data are presented as percentages or mean and SD. Comparative analyses were performed using the Student unpaired two-sided *T*-test, whereas the chi-square test was used for discrete variables. One-way analysis of variance (ANOVA) followed by Bonferroni’s post-hoc test were used to compare values between the different genotypes. Both univariate and multivariate Cox proportional hazard regression analyses as well as a Kaplan-Meier analysis were used to analyse and illustrate the risk of mortality during the follow-up period. The covariates that were adjusted for in the multivariate model were: Age, Hypertension, Ischemic heart disease, Body mass index, C-reactive protein, b-glucose, High density lipoprotein, Estimated glomerular filtration rate < 60 mL/min, Hemoglobin < 120 g/L, Ejection fraction < 40 % and N-terminal fragment of proBNP 4^th^ quartile. Both all-cause mortality and cardiovascular mortality were analysed. Censored patients were patients who were still alive at end of the study period or who had died of other causes than cardiovascular disease. Completed patients comprised those who had died due to cardiovascular disease.

A *P*-value < 0.05 was considered statistically significant. All data were analysed using standard software packages (Statistica v. 12.0, Statsoft Inc, Tulsa, OK, USA).

## Results

The basal characteristics of the total study population are presented in Table 1. Almost equal numbers of males and females were included (242 vs. 234). In the total study population, 359/476 (75.4 %) of the participants had hypertension, a significant greater part was females (79.1 % vs. 71.9 %). In the total study population 105/476 (22.1 %) had ischemic heart disease, and 10.7 % had anemia, defined as Hb > 120 g/L, where the female group was significantly overrepresented in comparison to the male group (15.0 % vs. 6.6 %). Significantly more males had impaired cardiac systolic function compared to the females (11.2 % vs. 3.8 %). The median follow-up time for the total population was 80 months (6.7 years), and during this time 106 all-cause and 62 cardiovascular deaths were registered.

**Table 1:** Basal characteristics of the total study population and divided into genders

Variables	Total population	Males	Females	*p*-value
N	476	242	234	
Age, mean (SD)	77.0 (3.4)	77.0 (3.2)	77.0 (3.7)	
BMI, (SD)	27.2 (4.3)	26.7 (3.3)	27.6 (5.1)	0.02
IHD, n (%)	105 (22.1)	63 (26.0)	42 (17.9)	0.03
Hypertension, n (%)	359 (75.4)	174 (71.9)	185 (79.1)	0.003
ACEI/ARB, n (%)	125 (26.3)	64 (26.4)	61 (26.1)	0.93
Hb < 120 g/L, n (%)	51 (10.7)	16 (6.6)	35 (15.0)	0.003
Beta blockers, n (%)	168 (35.3)	90 (37.2)	78 (33.3)	0.38
b-glucose, mmol/L, mean, (SD)	4.6 (1.3)	4.5 (1.2)	4.6 (1.4)	0.57
HDL, mmol/L mean, (SD)	1.5 (0.4)	1.3 (0.3)	1.6 (0.4)	<0.0001
eGFR < 60 mL/min, n (%)	233 (48.9)	116 (47.9)	117 (50.0)	0.65
CRP, mg/L, mean (SD)	11.0 (5.1)	11.0 (5.0)	11.0 (5.3)	0.68
EF < 40 %, n (%)	36 (7.6)	27 (11.2)	9 (3.8)	0.003
NT-proBNP ng/L, mean, (SD)	188 (319)	201 (395)	174 (200)	0.37
Adiponectin pg/mL, mean, (SD)	6263 (4690)	4829 (3391)	7884 (5387)	<0.0001

Notes: ACEI/ARB: Angiotensin converting enzyme inhibitor/Angiotensin receptor blocker; BMI: Body mass index; CRP: C-reactive protein; EF: Ejection fraction; eGRF: Estimated glomerular filtration rate; HDL; High density lipoproteins; IHD: Ischemic heart disease; NT-proBNP: N-terminal fragment of proBNP

### High-weight-molecular (HWM) adiponectin

The biologically active part of adiponectin, HWM-adiponectin was evaluated in the total study population. From the gender characteristics it could be seen that the female group had clearly higher concentration of adiponectin (mean: 7884 pg/mL vs. 4829 pg/mL; t = 6.51; *p* < 0.0001). The four quartiles of HMW-adiponectin (1^st^ quartile: < 3875 pg/mL; 2^nd^ quartile: 3875-6630 pg/mL; 3^rd^ quartile; 3876-8047 pg/mL; 4^th^ quartile; >8047 pg/mL) were evaluated according to cardiovascular risk.

### Adiponectin and cardiovascular mortality

In a univariate cox proportional hazard regression evaluating the prognostic information on cardiovascular mortality, it could be seen that those with high concentration of adiponectin had a significant increased risk (HR: 2.58 95 % CI 1.30–5.14; *p* = 0.007). This result persisted when evaluated adiponectin in a multivariate model including NT-proBNP, one of the most well-known biomarkers to evaluate cardiovascular risk (HR: 2.35 95 % CI 1.15–4.82; *p* = 0.02) (Table 2). A gender difference could be noted in the univariate Cox model (males: hazard ratio: 2.16; 95 % CI 0.52–9.07; *p* = 0.29 versus females: hazard ratio: 5.32; 95 % CI 1.93–14.71; *p* = 0.001). The gender difference previously described persisted, and in an interactions analysis a significant (*p* < 0.0001) interaction could be demonstrated. However, the limited sample size should be noted, and the interpretation should therefore be performed with caution

**Table 2:** Cox proportional hazard regression illustrating the risk of cardiovascular mortality during 6.7 years of follow-up of the study population having a high plasma concentration of adiponectin

Variables		Total population		Males			Females		
	Hazard ratio	95 % CI	*p*-value	Hazard ratio	95 % CI	p-value	Hazard ratio	95 % CI	*p*-value
Age	1.12	1.03–1.21	0.005	1.17	1.06–1.30	0.002	1.10	0.93–1.30	0.26
Hypertension	1.28	0.69–2.39	0.43	1.38	0.68–2.81	0.37	1.05	0.27–4,23	0.94
IHD	1.03	0.56–1.92	0.92	0.72	0.34–1.54	0.40	1.57	0.47–5.22	0.46
BMI	0.99	0.92–1.05	0.69	0.93	0.83–1.03	0.17	1.12	1.00–1.24	0.04
CRP	0.99	0.93–1.05	0.79	0.99	0.94–1.04	0.59	0.90	0.67–1.20	0.48
b-glucose	1.14	0.97–1.35	0.11	0.94	0.80–1.11	0.49	1.12	0.99–1.27	0.07
s-HDL	0.61	0.29–1.28	0.19	1.08	0.39–3.07	0.89	0.55	0.11–2.70	0.47
eGFR < 60 mL/min	0.72	0.34–1.55	0.40	0.66	0.28–1.55	0.34	0.78	0.12–5.19	0.79
Hb < 120 g/L	1.13	0.54–2.34	0.75	0.84	0.28–2.53	0.76	2.41	0.71–8.14	0.16
EF < 40 %	1.74	0.81–3.74	0.16	2.37	0.97–5.77	0.06	0.63	0.12–3.38	0.59
NT-proBNP Q4 (>197 ng/L)	2.54	1.21–5.34	0.01	1.62	0.67–3.89	0.28	6.30	1.36–29.14	0.02
Adiponectin Q4 (>12800 pg/mL)	2.35	1.15–4.82	0.02	1.66	0.38–7.26	0.50	5.28	1.59–17.57	0.007

Notes: BMI: Body mass index; CI: Confidence interval: CRP: C-reactive protein; EF: Ejection fraction; eGFR: Estimated glomerular filtration rate; HDL: High density lipoproteins; NT-proBNP: N-terminal fragment of proBNP; Q: Quartile

### Genotypes

The different genotypes C/C, C/G and G/G of rs266729 were analysed in the same population as evaluated above. In the total study population the C/C genotype was the most common in both the male and the female group (Table 3), followed by the C/G genotype. No gender difference could be seen regarding the distribution of genotypes of the rs266729.

**Table 3:** All-cause mortality in the study population distributed into the three analyzed genotypes of the adiponectin genes

	Females	Males	*p*-value
Adiponectin			
C/C, n (%)	130/234 (55.6)	133/242 (55.0)	*p* = 0.90
C/G, n (%)	84/234 (35.9)	96/242 (39.7)	*p* = 0.40
G/G, n (%)	20/234 (8.5)	13/242 (17.8)	*p* = 0.17

### Genotypes of rs266729 and cardiovascular mortality

In the total study population no increased cardiovascular mortality risk could be seen comparing the C/C carriers versus the C/G or the G/G carriers (HR: 1.54 95 % CI 0.91–2.61; *p* = 0.11) (Table 4). However, evaluating the different genders the female group exhibited in a univariate Cox proportional hazard regression analysis a highly increased risk of cardiovascular mortality in the rs266729 C/C genotype compared to the C/G or G/G genotypes (HR: 6.48; 95 % CI 1.49–28.18; *p* = 0.01), although the confidence interval was wide. Performing a multivariate analysis of the Cox proportional hazard regression, where risk of cardiovascular mortality was evaluated, a highly significant increased risk was demonstrated (HR: 9.35; 95 % CI 1.99–43.91; *p* = 0.005), even in competition with clinical variables influencing cardiac risk (Table 4). Again, the confidence interval is wide and the result should therefore be interpreted with caution. No prognostic information could be demonstrated in the male group (HR: 0.87; 95 % CI 0.47–1.62; *p* = 0.66), and an interaction analysis supported the gender difference reported (*p* = 0,000082).

**Table 4:** Cox proportional hazard regression illustrating the risk of cardiovascular mortality in the C/C allele versus the C/G or G/G allele of rs266729 SNP of adiponetin adjusted for well-known clinical risk factors influencing cardiovascular risk in an elderly population during 80 months of follow-up

Variables		Total population		Males			Females		
	Hazard ratio	95 % CI	p-value	Hazard ratio	95 % CI	p-value	Hazard ratio	95 % CI	p-value
Age	1,13	1.05-1.22	0.002	1.18	1.06-1.30	0.001	1.16	0.99-1.34	0.06
Hypertension	1.34	0.71-2.51	0.36	1.42	0.68-2.95	0.35	1.50	0.36-6.22	0.58
IHD	1.15	0.63-2.10	0.64	0.77	0.36-1.64	0.50	1.92	0.63-5.79	0.25
BMI	0.98	0.92-1.05	0.60	0.92	0.83-1.02	0.11	1.08	0.98-1.19	0.14
CRP	1.0	0.94-1.06	0.90	0.99	0.94-1.04	0.61	0.89	0.65-1.20	0.44
b-glucose	1.12	0.96-1.32	0.16	1.10	0.90-1.34	0.34	1.17	0.85-1.61	0.34
HDL	0.68	0.33-1.40	0.29	1.19	0.40-3.48	0.76	0.78	0.23-2.64	0.68
eGFR<60mL/min	1.25	0.71-2.20	0.43	0.85	0.43-1.70	0.65	4.24	1.08–16.60	0.04
Hb<120g/L	1.18	0.57-2.45	0.65	0.87	0.30-2.58	0.80	2.14	0.70-6.57	0.18
EF<40 %	1.78	0.84-3.77	0.13	2.40	0.97-5.97	0.06	2.45	0.48-12.36	0.28
Rs266729 C/C vs. C/G or G/G	1.54	0.91-2.61	0.11	0.87	0.47-1.62	0.66	9.35	1.99-43.91	0.005

Note: BMI: Body mass index; CRP; C-reactive protein; eGFR: Estimated glomerular filtration rate; EF: Ejection fraction; HDL; High density lipoprotein

**Fig. 1:**
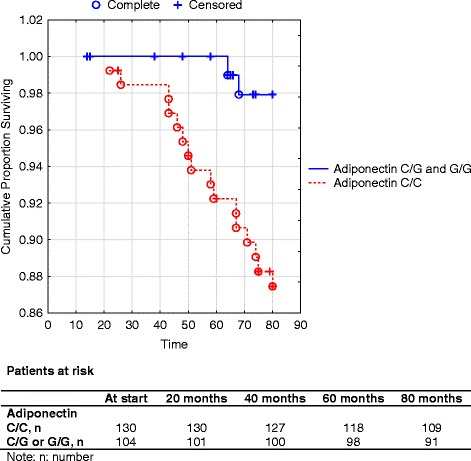
Cumulative proportion surviving from cardiovascular mortality as reflected by the adioponectin genotypes C/C vs. C/G or G/G in an elderly female population followed during 80 months. Note: Censored participants were those still living at the end of the study period, or those who had died for reasons other than cardiovascular disease. Completed participants were those who had died due to cardiovascular disease

A Kaplan-Meier analysis showed that C/C carriers of the rs266729 gene exhibited higher cardiovascular mortality compared to the C/G or the G/G genotypes in the female group (Fig. 1). However, the sample size is limited.

Performing the corresponding analyses of the different genotypes of rs266729 in the male group did not reveal any significant differences between the three genotypes.

## Discussion

### Adiponectin and cardiovascular mortality

The protein adiponectin has a multifaceted action in the body, including effects in the cardiovascular area. It is generally believed that adiponectin suppresses apoptosis, and directly protects the cardiac cells from oxidative stress and inflammation and finally from ischemic injuries [28]. The complexity of actions of adiponectin is illustrated by the fact that even if adiponectin have a cardiovascular protecting effect [6], many epidemiological studies have shown that high levels is associated with increased cardiovascular mortality [9, 10, 29, 30]. The data we present gives support for this observation with higher mortality in those with high plasma level of of the SNP rs266729 of adiponectin.

Therefore, the hypothesis has been launched that a healthy and a diseased population differs in pattern of reaction on high levels of adiponectin [31], and there are reports indicating a cardioprotective effect of high levels of adiponectin in healthy individuals [32]. Furthermore, in the literature there are reports indicating a gender difference in serum levels of adiponectin where the females have higher levels compared to males [8, 30]. We could corroborate this relation as we can report a highly significant difference between the two genders with a higher plasma level in the females.

We have evaluated the biologically active part of adiponectin, the high molecular weight part. Even if the vast majority of reports in the literature is on total adiponectin measurements, the results regarding cardiovascular risk evaluations can be compared, as reported by Karas et al. [33]. We could demonstrate a more than 2 fold increased risk of cardiovascular mortality if high levels of HMW-adiponectin could be measured, even in competition with NT-proBNP, and when adjusted for other well-known clinical variables. Even if the confidence interval is wide, the information that high levels of adiponectin is a significant risk, is robust.

The golden standard of biomarkers in the handling of heart failure is NT-proBNP, and in the literature there are reports indicating a significant correlation between NT-proBNP and plasma adiponectin in asymptomatic older men [34]. Wannamethee et al. reported a correlation coefficient of 0.16 (*p* < 0.0001) [34]. We could report the same correlation coefficient in the corresponding evaluation in our male population (*r* = 0.16; *p* = 0.024), and an even higher correlation coefficient in our female population (*r* = 0.22; *p* = 0.008).

### Genotypes of rs266729 and cardiovascular mortality

For the first time we have also investigated the distribution and the prognostic information of the three genotypes C/C, C/G and G/G of rs266729 SNP of adiponectin in the same population as evaluated above. We could report a higher cardiovascular risk for the C/C carrier compared to the two other genotypes among the females, but not among the males, even if the gender difference should be interpreted with caution due to a small sample size. As there are variables that are associated with the rs266729 SNP, as systolic blood pressure reported by Avery et al. we have adjusted for hypertension in our multivariate evaluations, and still could report the significant difference in cardiovascular risk of the three genotypes in females [35]. As there is an association between b-glucose and adiponectin, and between high density lipoprotein, adjustments have also been done with these covariates. The result, however, persisted.

The two genders have been evaluated regarding cardiovascular risk in those with high adiponectin concentration, and the C/C carriers. Even after adjusting for multiple testing using Bonferroni’s rule (multiplying the obtained *P* value with the number of tests performed), there is still a clear significance for the adjusted *P* value obtained in the female group (*p* = 0.028 for the increased cardiovascular risk in those with a adiponectin concentration in the 4^th^ quartile, and *p* = 0.002 for the increased cardiovascular risk of the female C/C carriers in the multiple models).

The presented study has a limited sample size influencing the obtained results of the statistical evaluations. Therefore, it does not have power to exclude that the male group also could have increased risk in those with high adiponectin concentration, or are C/C carriers, as could be seen from the relatively wide confidence intervals of the obtained point estimate in the multiple Cox regressions. However, the results presented indicate that for those with high adiponectin levels, or for the C/C carriers an increased risk exist.

The obtained results are interesting, and as the cost for the obtained genetic evaluations are not much higher than that from biomarker analyses done in the clinical routine, it could be suggested that this type of evaluation might help the clinician to identify a group with increased cardiovascular risk when health resources are limited.

### Limitations

The presented research is a community-based study, and thus the majority of patients had discrete or no cardiovascular symptoms. However, those with cardiovascular disease (ischemic heart disease and/or hypertension and/or diabetes and/or heart failure: were not in the minority (419 out of 476: 88 %) and thus the size of those with increased cardiac risk is significant. However, the total study population was small which resulted in wide confidence intervals in risk evaluations, thus making the interpretation of these uncertain.

Also, the presented study population was an elderly population, and therefore it was not possible to extrapolate the obtained results into other age groups. Besides, this population consists of an elderly Swedish population consisting of only Caucasian participants. It is therefore not possible to extrapolate the results into other ethnicities.

Also, due to the limited sample size the gender differences demonstrated should be regarded as hypothesis-generating, and the interpretation should therefore be drawn with caution, even if the interaction analysis point to a specific gender difference.

Finally, as the subgroups of those with certain genotypes are small, the results of some of the evaluations should be interpreted with caution. The presented study should therefore be regarded as hypothesis generating, and should warrant for further research.

## Conclusion

Adiponectin is a protein that have complex functions within the body. We present data indicating an association between high plasma levels of adiponectin and increased cardiovascular risk for elderly healthy persons, and there is indications that there is a gender difference. We could also present that in the same population the females with the C/C carrier of the rs266729 SNP of adiponectin had a significantly increased cardiovascular risk compared to the C/G or the G/G carriers. No such a difference in cardiovascular risk could be observed in the males.

We suggest that these data, as extracted from a relatively small sample would be regarded as hypothesis- generating, and to warrant further research in this expanding area.
